# Supplementary material for: Improvements in task performance after practice are associated with scale-free dynamics of brain activity
Source: Netw Neurosci. 2023 Oct 1;7(3):1129–52. doi: 10.1162/netn_a_00319 (PMC10473260; doi:10.1162/netn_a_00319)
Supplement: Supplementary file 1 [file netn-7-3-1129-s001.docx]

**Supplementary Results**

**Title:** Improvements in task performance after practice are associated with scale-free dynamics of brain activity

**Authors:** Omid Kardan^1,2^, Andrew J. Stier^1^, Elliot A. Layden^1^, Kyoung Whan Choe^1^, Muxuan Lyu^3^, Xihan Zhang^1^, Sian L. Beilock^4^, Monica D. Rosenberg^1^, & Marc G. Berman^1^

^1^University of Chicago, Chicago, IL, USA

^2^University of Michigan, Ann Arbor, MI, USA

^3^The Hong Kong Polytechnic University, Hong Kong

^4^Barnard College, Columbia University, NY, USA

Address correspondence to Omid Kardan [omidk@med.umich.edu](mailto:omidk@med.umich.edu), or Marc G. Berman [bermanm@uchicago.edu](mailto:bermanm@uchicago.edu)

**Supplementary section 1. PLS results from a different parcellation (Craddock 392).**

To assess the stability of the PLS results associating *H* in brain parcels to improvements in task performance, we repeated the analyses with a different whole-brain brain parcellation consisting of 392 parcels (Craddock et al., 2012). Across all three datasets, we found consistent PLS results with the Shen 268-node parcellation scheme. For each analysis, *H* positively loaded on higher task-performance improvements, i.e., increases in H were related to improvements in task performance. The stability of the spatial brain patterns was low to moderate (see below for details). Specifically, we correlated the brain LV1 from Craddock parcels and Shen parcels in each PLS by up-sampling both versions to the common voxel space, and tested the correlation between the two patterns against correlations between up-sampled permuted brain LV values (1000 permutations).

Dual n-back task PLS results: In the DNB task, we found a pattern of higher *H* across brain parcels (using Craddock 392 parcels) which was related to greater improvement in the dual n-back task from run1 to run2, adjusted for performance in run1 (adj. ΔA’). The spatial pattern in the brain *H* latent variable was moderately correlated with the original analysis (i.e., Shen 268-node parcellation scheme) when projected back into voxel space (r = .375, p<.001). This shows that the granularity of the brain parcellation impacts the spatial pattern of the brain latent variable associated with performance improvement in the PLS, but the general direction is unchanged (i.e., higher *H* is related to performance improvement). Figure S1 shows the PLS result for the Craddock 392-node parcellation in the dual n-back study.

**
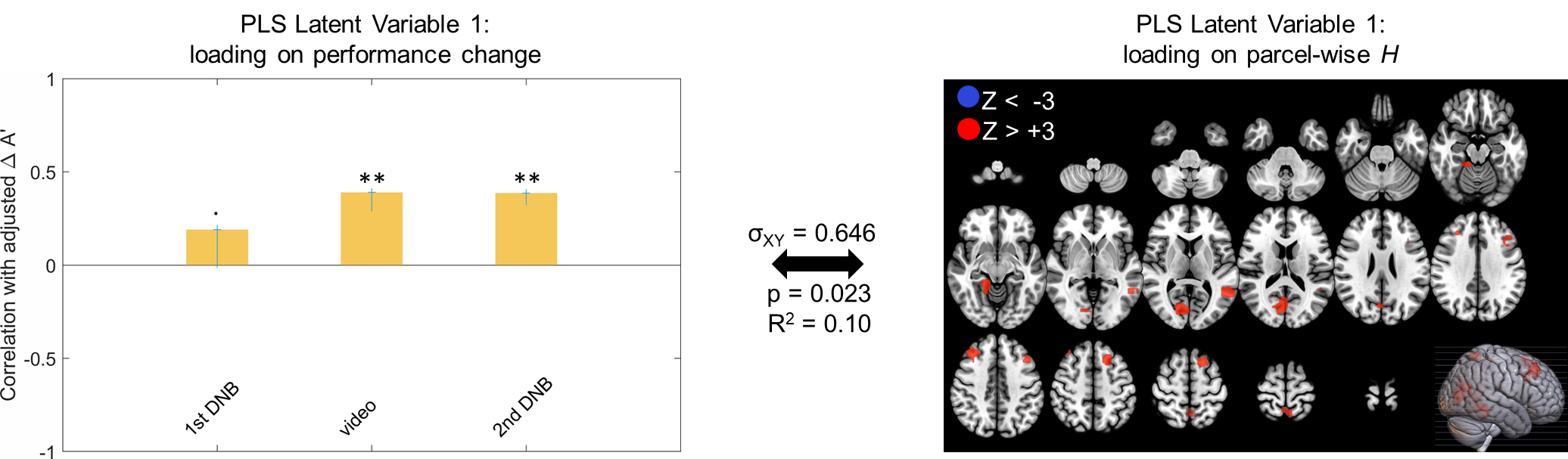
**

**Figure S1.** The primary latent variable from Behavioral PLS relating adj. ΔA’ to parcel-wise *H* in the DNB experiment with Craddock 392 parcels. All red parcels (total of 8) in the right panel show Bootstrap ratio Z_BR_ values above +3 and there are no blue parcels with Z_BR_ < −3, indicating an exclusively positive direction for the H-to-adj. ΔA’ association. Cross-block covariance (σ_XY_) shows the proportion of covariance between the left and right panel explained by this LV, and the p-value is calculated from a permutation test for the eigenvalue for this LV.

N-back task PLS results: In the HCP dataset, we again found a pattern of higher *H* using the Craddock 392 brain parcels which was related to greater improvement in the n-back task from run1 to run2, adjusted for performance in run1 (adj. ΔAccuracy). The spatial pattern in the brain *H* latent variable was moderately correlated with the LV from the original Shen 268-node analysis (r = .478, p<.001). This shows that the granularity of the brain parcellation moderately impacts the spatial pattern of the brain latent variable associated with performance improvement in the PLS, but the general direction is unchanged (i.e., higher *H* is related to performance improvement). Figure S2 shows the PLS result for the Craddock 392-node parcellation in the n-back study.

**
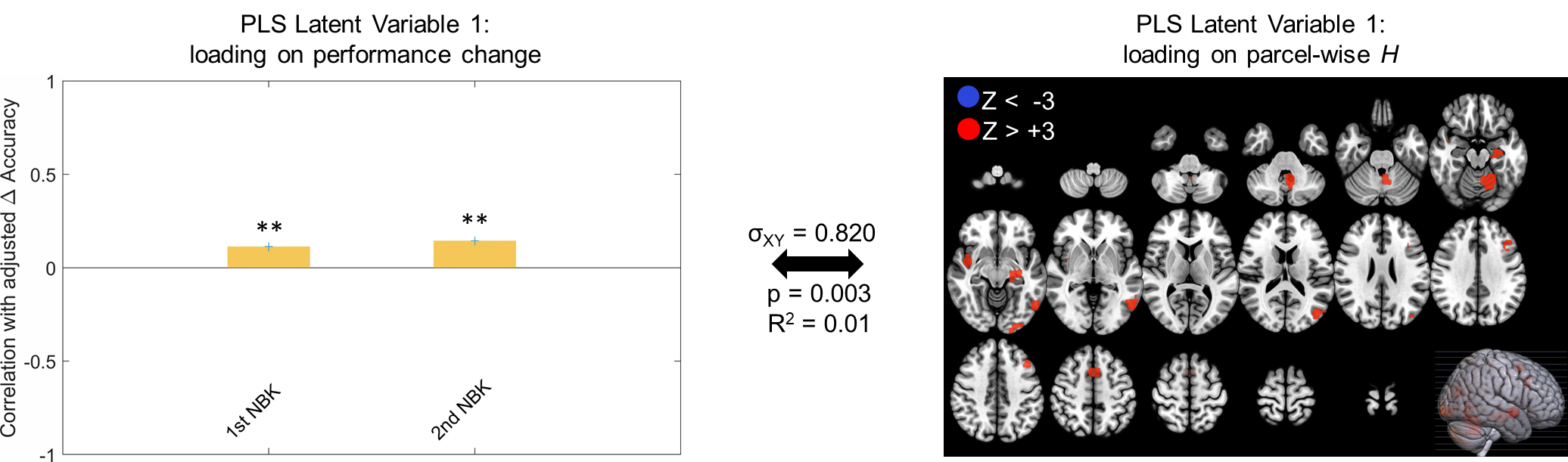
**

**Figure S2.** The primary latent variable from Behavioral PLS relating adj. ΔAccuracy in the n-back task to parcel-wise *H* with Craddock 392 brain parcels. All red parcels (total of 9) in the right panel show Bootstrap ratio Z_BR_ values above +3 and there are no blue parcels with Z_BR_ < −3, indicating an exclusively positive direction for the H-to-adj. ΔA’ association. Cross-block covariance (σ_XY_) shows the proportion of covariance between the left and right panel explained by this LV, and the p value is calculated from permutation test for the eigenvalue for this LV.

Choose-and-Solve Task (CAST) PLS results: In the CAST dataset, we found a general pattern of greater task improvement associated with higher *H* in the PLS using Craddock 392 brain parcels, although one brain parcel with negative association also emerged (i.e., Z_BR_ < -3) in this analysis (compared to 4 parcels with Z_BR_ > +3). At less stringent threshold of |Z| > 2, there were 24 parcels with Z_BR_ > +2 compared to 5 parcels with Z_BR_ < -2. The spatial pattern in the brain *H* latent variable had a small but significant correlation with the LV in the original Shen 268-node analysis r = .174, p<.001). This again shows that the granularity of the brain parcellation impacts the spatial pattern of the brain latent variable associated with performance improvement in the PLS, but the general direction is consistent (i.e., higher *H* is related to performance improvement). The spatial pattern was more impacted here compared to the other two tasks, which may be due, in part, to the higher number of possible latent variables in the PLS (6 LVs) and smaller sample size. Figure S3 shows the PLS result for the Craddock 392-node parcellation in the CAST study.

**
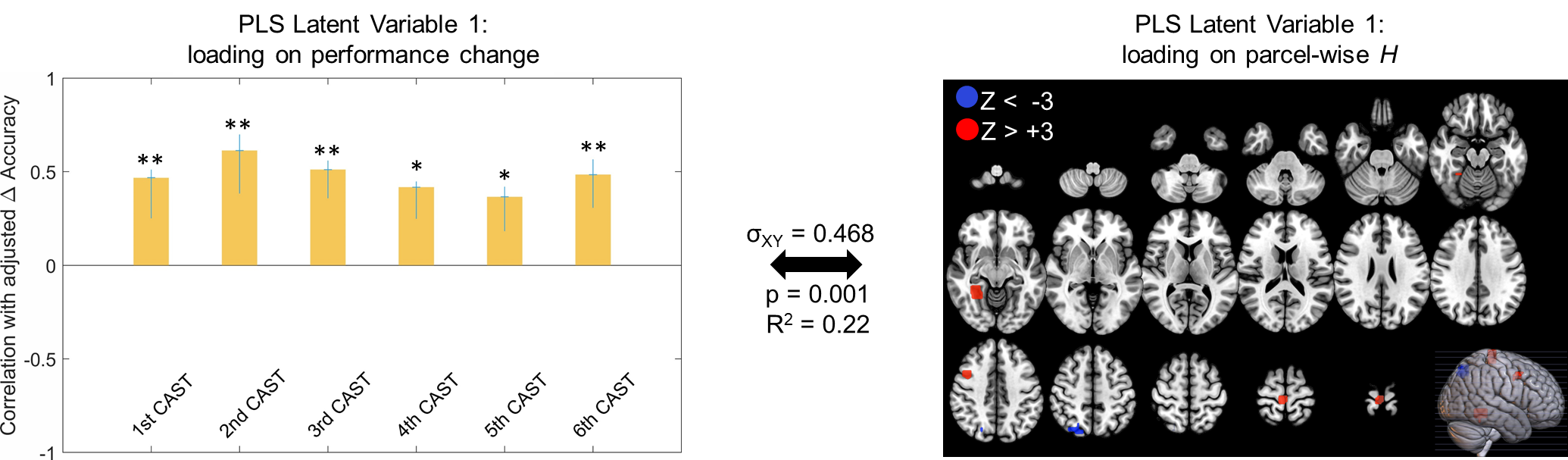
**

**Figure S3.** The primary latent variable from Behavioral PLS relating adj. ΔAccuracy in the CAST task to parcel-wise *H* with Craddock 392 brain parcels. Red parcels (total of 4) in the right panel show Bootstrap ratio Z_BR_ values above +3 and one blue parcel with Z_BR_ < −3 shows negative association with the left panel (Δaccuracy). Cross-block covariance (σ_XY_) shows the proportion of covariance between the left and right panel explained by this LV, and the p value is calculated from a permutation test for the eigenvalue for this LV.

**Supplementary section 2. Results are robust to block-level temporal structure of task fMRI runs.** To assess if differences in the temporal structures of the tasks contributed to the reported findings, we recalculated the H exponents in the three datasets after regressing out the temporal block structure in each task run from the BOLD timeseries. Note that contributions from trial-level temporal structure are too fast and are not included in the frequency range of our fMRI data. Therefore, in this analysis we only removed dynamics that are slower than ~8 seconds (i.e., block changes) corresponding to the .12 Hz upper bound of the band-pass filtered fMRI data. Block-timing regressors were vectors of 0 and 1 corresponding to the onset and offset of task blocks convolved with the default double-gamma function of Statistical Parametric Mapping (SPM-12) for the hemodynamic response function (HRF).

Dual n-back task:

First, we compared the calculated mean *H* values for each participant per run between the original and block-time-regressed analyses. The average *H* over brain parcels after block timing regression were highly correlated with the original analysis across participants in the DNB dataset (Pearson *r*s > .955, *p*s <.001 for both DNB runs). Our finding that the overall *H* mean was higher during the video run in the DNB study compared to the two DNB task runs was also replicated in this version of the analysis where the temporal structure of the DNB tasks were regressed out (t(55) = 13.69, p < .001). Next, we assessed the PLS analyses. The new results replicated the PLS results without the block-structure regressors from Figure 3. Specifically, a pattern of higher *H* across the brain was related to greater improvement in the dual n-back task from run1 to run2, adjusted for performance in run1. The spatial pattern in brain H latent variable was highly correlated with the original analysis (r = .952, p<.001). The Z-thresholded maps also looked similar as shown in Figure S4, (3 parcels had Z>+3, 0 parcels had Z<-3; all of these 3 parcels were among the 5 parcels with Z>+3 in the original dual n-back PLS results in Fig 3).


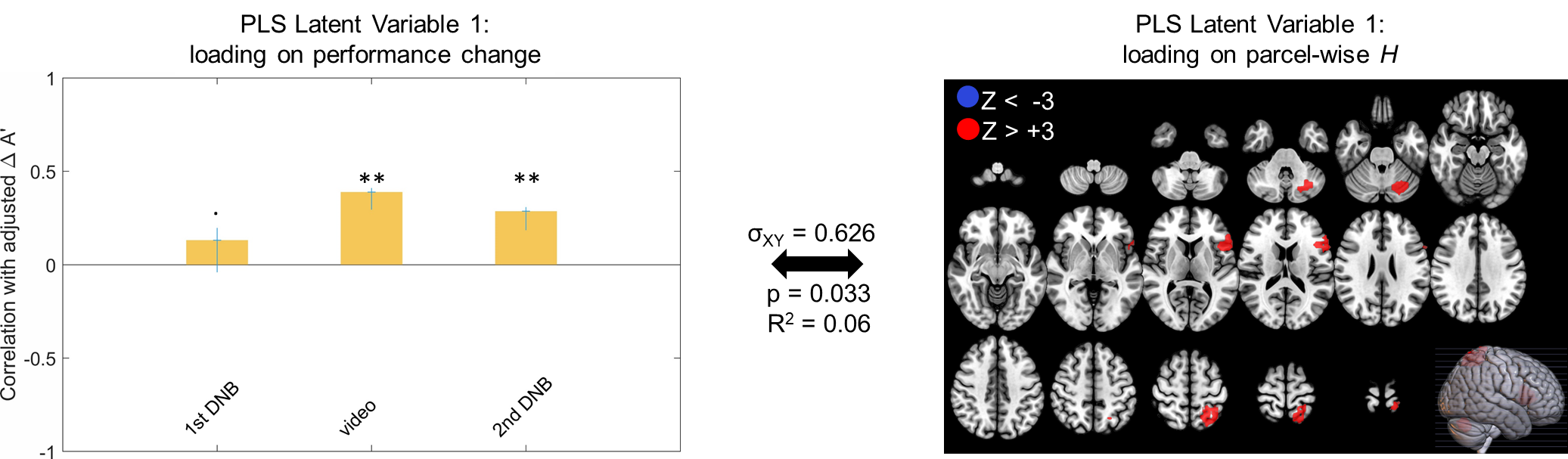


**Figure S4.** The primary latent variable from Behavioral PLS relating adj. ΔA’ to parcel-wise *H* in the DNB experiment with the temporal structure of the task blocks regressed out of the parcel timeseries prior to calculation of *H* values. All red parcels (total of 3) in the right panel show Bootstrap ratio Z_BR_ values above +3 and there are no blue parcels with Z_BR_ < −3, indicating exclusively positive direction for the H-to-adj. ΔA’ association. Cross-block covariance (σ_XY_) shows the proportion of covariance between the left and right panel explained by this LV, and the p value is calculated from a permutation test for the eigenvalue for this LV.

N-back task:

In the HCP dataset, first, we compared the calculated mean *H* values for each participant per run between the original and block-time-regressed analyses. The average *H* over brain parcels after block timing regression were highly correlated with the original analysis across participants in the HCP dataset (Pearson *r*s > .984, *p*s <.001 for both NBK runs). Next, we assessed the PLS analyses. The results replicated the PLS results without the block-structure regressors from Figure 4. Specifically, a pattern of higher *H* across the brain was related to more improvement in the n-back task from run1 to run2, adjusted for performance in run1. The spatial pattern in brain *H* latent variable was highly correlated with the original analysis (r = .975, p<.001). The Z-thresholded maps also look very similar to those in the version without block timing regressors as shown in Figure S5, (8 parcels had Z>+3, 0 parcels had Z<-3; all of these 8 parcels were among the 9 parcels with Z>+3 in the original n-back PLS results in Fig 4).


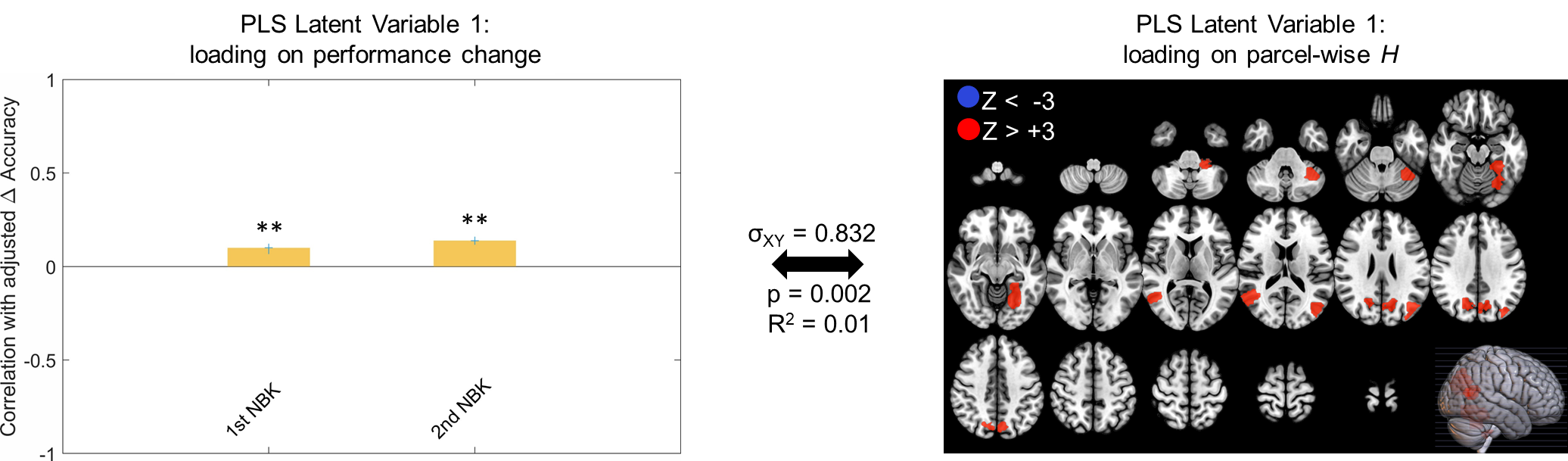


**Figure S5.** The primary latent variable from Behavioral PLS relating adj. ΔAccuracy in the n-back task to parcel-wise *H* with task block timings regressed out of the timeseries prior to calculating *H* values. All red parcels (total of 8) in the right panel show Bootstrap ratio Z_BR_ values above +3 and there are no blue parcels with Z_BR_ < −3, indicating exclusively positive direction for the H-to-adj. ΔAccuracy association. Cross-block covariance (σ_XY_) shows the proportion of covariance between the left and right panel explained by this LV, and the p value is calculated from a permutation test for the eigenvalue for this LV.

Choose-and-solve task (CAST):

In the third study, we first compared the calculated mean *H* values for each participant per run between the original and block-time-regressed analyses. The average *H* over brain parcels after block timing regression were highly correlated with those without the timing regressors across participants (Pearson *r*s > .987, *p*s <.001 for all 6 runs of CAST). Next, we assessed the PLS analysis. The results replicated the PLS results without the block-structure regressors from Figure 5. Specifically, a pattern of higher *H* across the brain was related to greater improvement in the CAST task from run1 to run6, adjusted for performance in run1. The spatial pattern in the brain *H* latent variable was highly correlated with the original analysis (r = .990, p<.001). The Z-thresholded maps were the same between the two analyses as shown in Figure S6, (4 parcels had Z>+3, 0 parcels had Z<-3; all of these 4 parcels were the same as the original CAST PLS results in Fig 5).


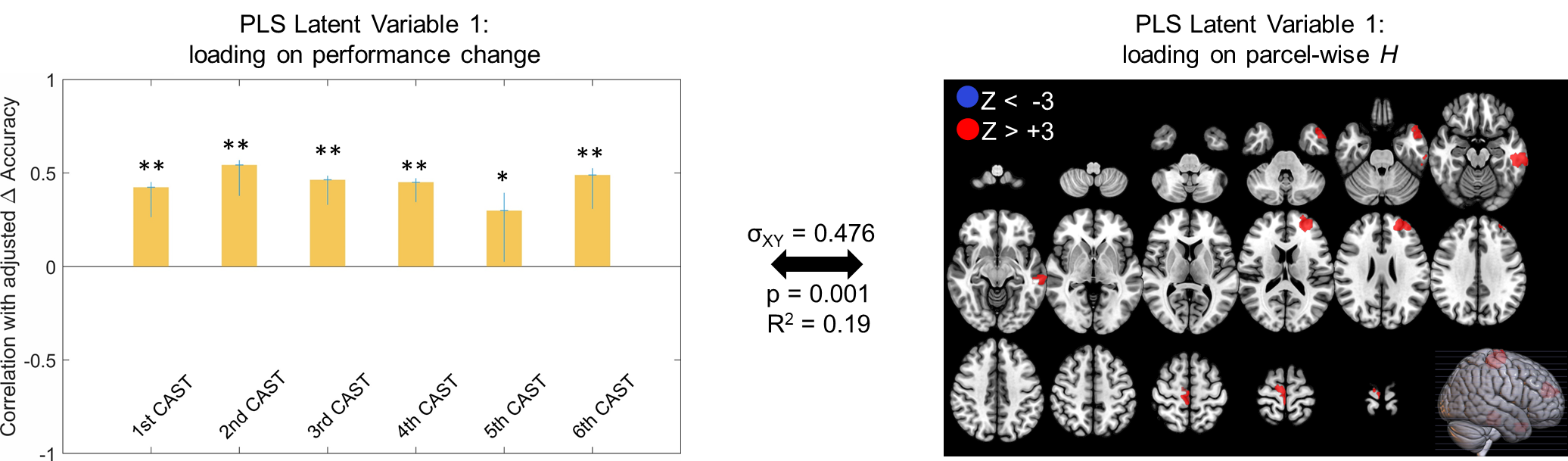


**Figure S6.** The primary latent variable from Behavioral PLS relating adj. ΔAccuracy in the CAST task to parcel-wise *H* with task block timings regressed out of the timeseries prior to calculating *H* values. All red parcels (total of 4) in the right panel show Bootstrap ratio Z_BR_ values above +3 and there are no blue parcels with Z_BR_ < −3, indicating exclusively positive direction for the H-to-adj. ΔAccuracy association. Cross-block covariance (σ_XY_) shows the proportion of covariance between the left and right panel explained by this LV, and the p value is calculated from a permutation test for the eigenvalue for this LV.

**Supplementary section 3. Linear fit of the single *H* exponents to the data range.** To assess if our single *H* exponents were a good fit to the range of scales in the data, we calculated the R^2^ of the linear fit between F(n) (i.e., fluctuations) and n (i.e., window size) from the DFA in each brain parcel of each run for each participant. Overall, we found a very good linear fit of the de-trended fluctuation variance as a function of temporal scale across the datasets (examples from each task are shown in Figures S7-S9 below).

H exponent fit in dual n-back task: We calculated the R^2^ values for the regression of log(n) on log(F(n)) in the DNB dataset (dual n-back task and video). The linear fit was a good fit to these data, with R^2^ values in the range of min R^2^ = .720 to max R^2^ = .997 across all brain parcels and participants. Figure S7 shows some examples for two random participants across runs (each line is a brain parcel).


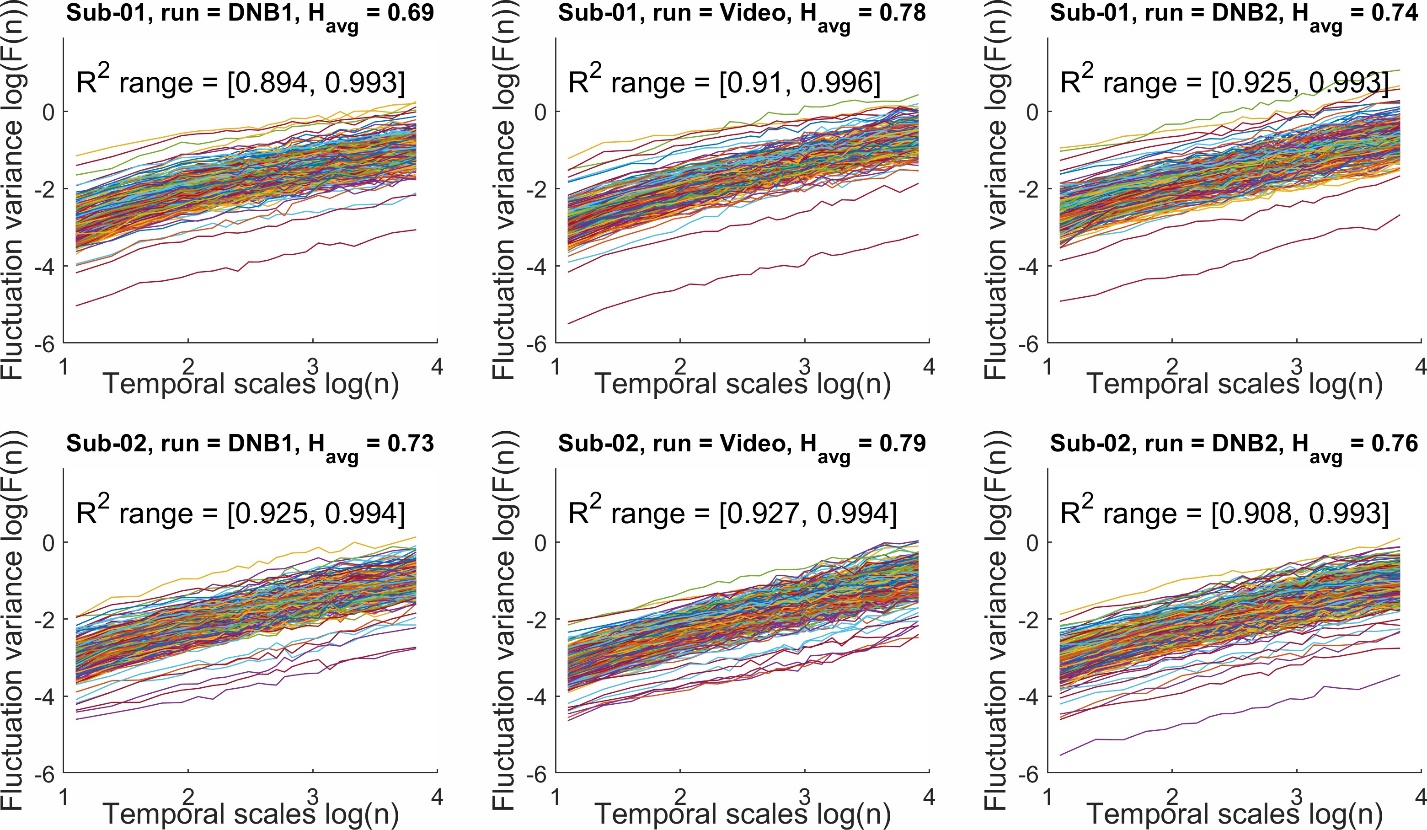


**Fig S7.** Examples of DFA fits to the fMRI data at the parcel level in the dual n-back study for two random participants. Top row panels show subject 1 across the three runs and bottom row panels show subject number 2 across the 3 runs. Each line represents a brain parcel (from the Shen 268-node atlas).

H exponent fit in the n-back task: We also calculated the R^2^ values for the regression of log(n) on log(F(n)) in the Human Connectome dataset (n-back task). The linear fit was good to these data, with R2 values in the range of min R^2^ = .892 to max R^2^ = .997 across all brain parcels and participants.

**
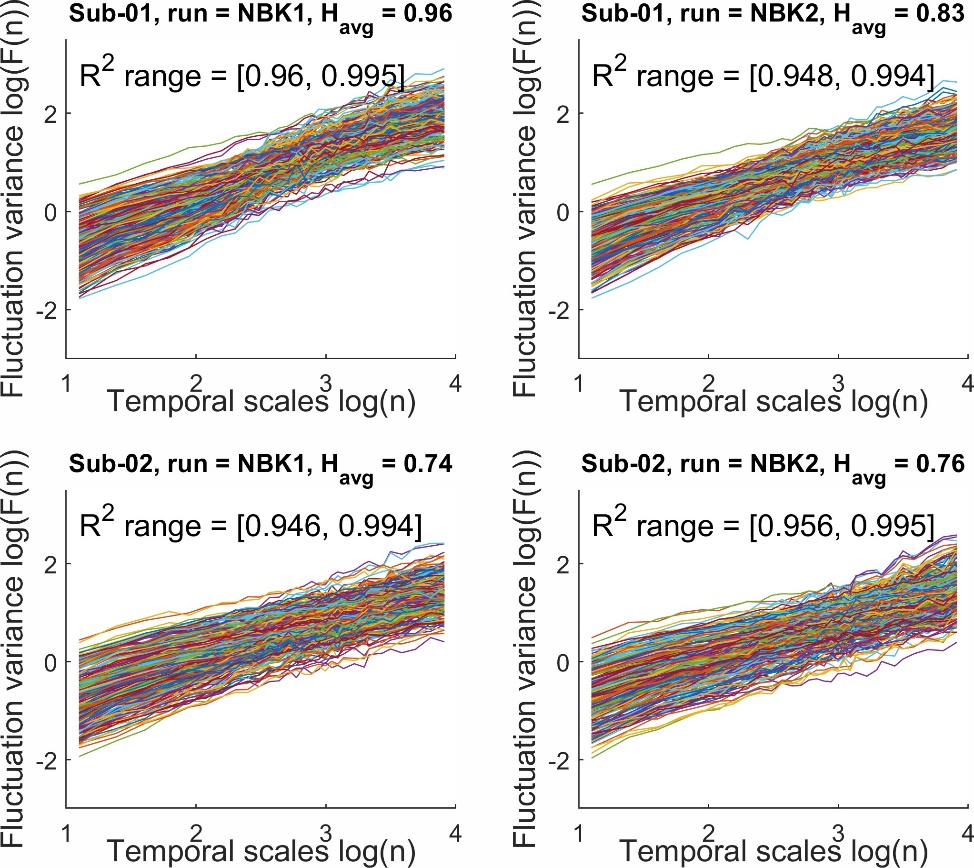
**

**Fig S8.** Examples of DFA fits to the fMRI data at the parcel level in the n-back study (HCP dataset) for two random participants. Top row panels show subject 1 across the n-back runs and bottom row panels show subject 2 across the n-back runs. Each line represents a brain parcel (from the Shen 268-node atlas).

H exponent fit in the CAST task: We also calculated the R^2^ values for the regression of log(n) on log(F(n)) in the Study 3 dataset (choose-and-solve task). Again, the linear fit was good to these data, with R^2^ values in the range of min R^2^ = 729 to max R^2^ = 996 across all brain parcels and participants.

**
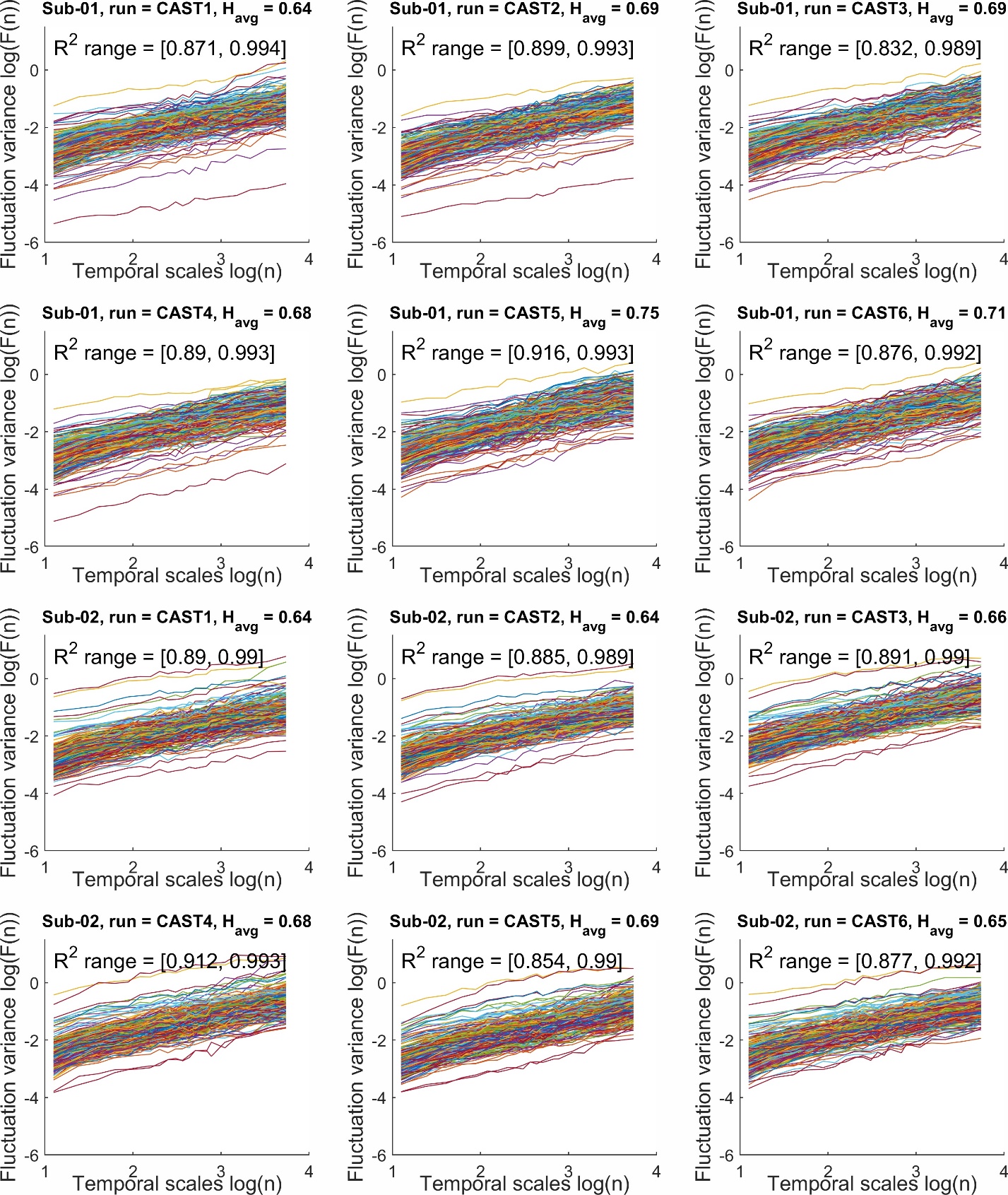
**

**Fig S9.** Examples of DFA fits to the fMRI data at the parcel level in the CAST study for two random participants. Top two rows show subject 1 across the six CAST runs and bottom two rows show subject 2 across the CAST runs. Each line represents a brain parcel (from the Shen 268-node atlas).

**Supplementary section 4. Wavelet Leaders Multifractal (WLMF) analysis**

The wavelet leader multifractal (WLMF) formalism has emerged as a powerful technique to estimate *H* that is highly robust to signal non-stationarity (Jaffard et al., 2007). To analyze a signal of interest at different delays and timescales, the wavelet transform uses translated and dilated versions of a basis function $\Psi\left( \left[ t-k \right]/a \right)$. Specifically, signal energy present at delay *k* and at time scale *a* is the wavelet coefficient $d_{x}\left( a,k \right)$ which is measured by calculating the integral $d_{x}\left( a,k \right)=\frac{1}{a}\int x(t)\Psi\left( \frac{t-k}{a} \right)dt$, where $a=2^{j}$ for integer *j* represents a range of dyadic scales. Wavelet leaders $L_{x}\left( a,k \right)$ are subsequently calculated as the largest coefficient value $\left| d_{x}\left( a',k' \right) \right|$ within a narrow temporal neighbourhood of *k*, for any scale $a'\leq a$. Multifractal scaling is then defined by the function $\frac{1}{K}\sum_{k} \left| L_{x}\left( 2^{j},k \right) \right|^{q}=C_{q}2^{j\varsigma(q)}$ which describes wavelet power as a function of time scale, for a range of different scaling exponents *q*, in terms of a characteristic function $\varsigma(q)$. To assess linear, quadratic and cubic components of the scaling function, $\varsigma(q)$ was parameterized as a polynomial expansion $\varsigma\left( q \right)=\sum_{p} c_{p}\left( q^{p}/p! \right)$, where the log-cumulants *c*_p_ define the scaling behavior of the signal $x(t)$. In our first analysis, we focused on first-order cumulant *c*_1_, which is closely linked to the monofractal scaling parameter *H* from DFA (Wendt, et al, 2007). These yielded similar results showing higher H (c1) across brain parcels was related to greater task performance improvement across the three datasets. In our second analysis we assessed the higher-order cumulants (*c*_2_ and *c*_3_) and found them to have values close to zero, demonstrating a lack of systematic non-linear scaling in these data.

**Supplementary section 4.1. Comparison of DFA results with WLMF first cumulant.**

Dual n-back task PLS results: Using the first cumulant (*c1*) of the WLMF to estimate the Hurst Exponent (*H*) instead of DFA yielded similar results in the PLS. Specifically, a pattern of higher *c1* across the brain was related to greater improvement in the dual n-back task from run1 to run2, adjusted for performance in run1. The spatial pattern in brain *H* (*c1)* for latent variable 1 was highly correlated with the original *H* (DFA) analysis (r = .647, p<.001). The Z-thresholded maps for brain *c1* latent variable are shown in Figure S10, (5 parcels had Z>+3, 0 parcels had Z<-3; one of these 5 parcels were among the 5 parcels with Z>+3 in the DFA-based original dual n-back PLS result in Fig 3).


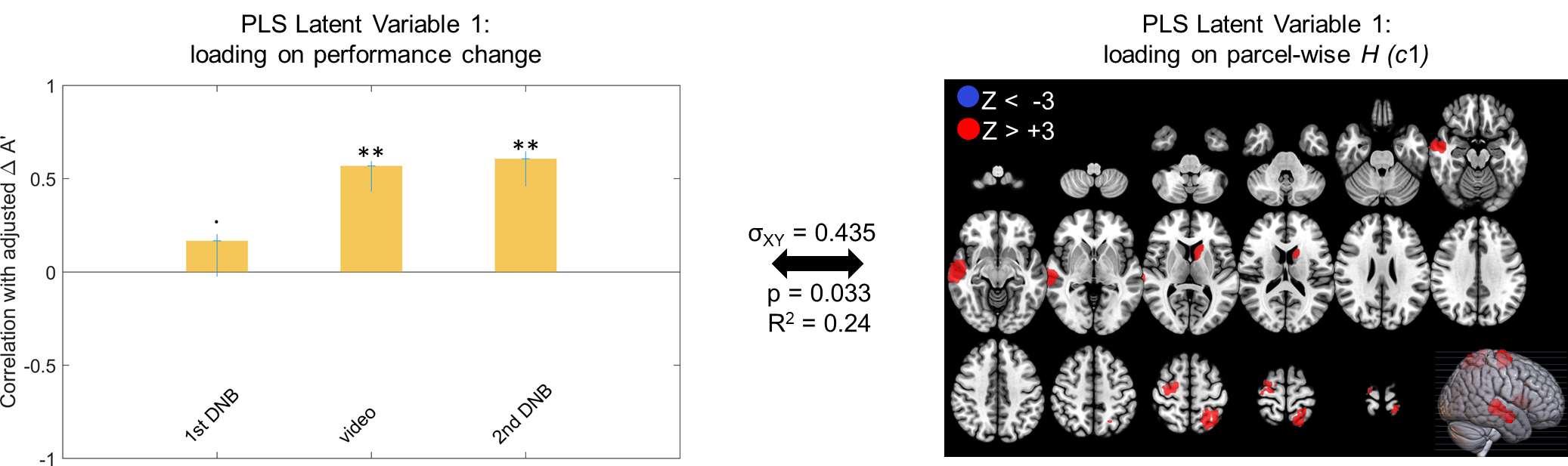


**Figure S10.** The primary latent variable from Behavioral PLS relating adj. ΔA’ to parcel-wise *H* in the DNB experiment with *H* values estimated as *c*1 in WLMF analysis. All red parcels (total of 5) in the right panel show Bootstrap ratio Z_BR_ values above +3 and there are no blue parcels with Z_BR_ < −3, indicating exclusively positive direction for the H-to-adj. ΔA’ association. Cross-block covariance (σ_XY_) shows the proportion of covariance between the left and right panel explained by this LV, and the p value is calculated from a permutation test for the eigenvalue for this LV.

N-back task PLS results: Using the first cumulant (*c1*) of the WLMF to estimate the Hurst Exponent (*H*) instead of DFA yielded similar results in the PLS for the HCP dataset. Specifically, a pattern of higher *c1* across the brain was related to more improvement in the n-back task from run1 to run2, adjusted for performance in run1. The spatial pattern in brain *H* (*c1)* for latent variable 1 was highly correlated with the original *H* (DFA) analysis (r = .757, p<.001). The Z-thresholded maps for brain *c1* latent variable are shown in Figure S11, (9 parcels had Z>+3, 0 parcels had Z<-3; four of these 9 parcels were among the 9 parcels with Z>+3 in the DFA-based original n-back PLS result in Fig 4).


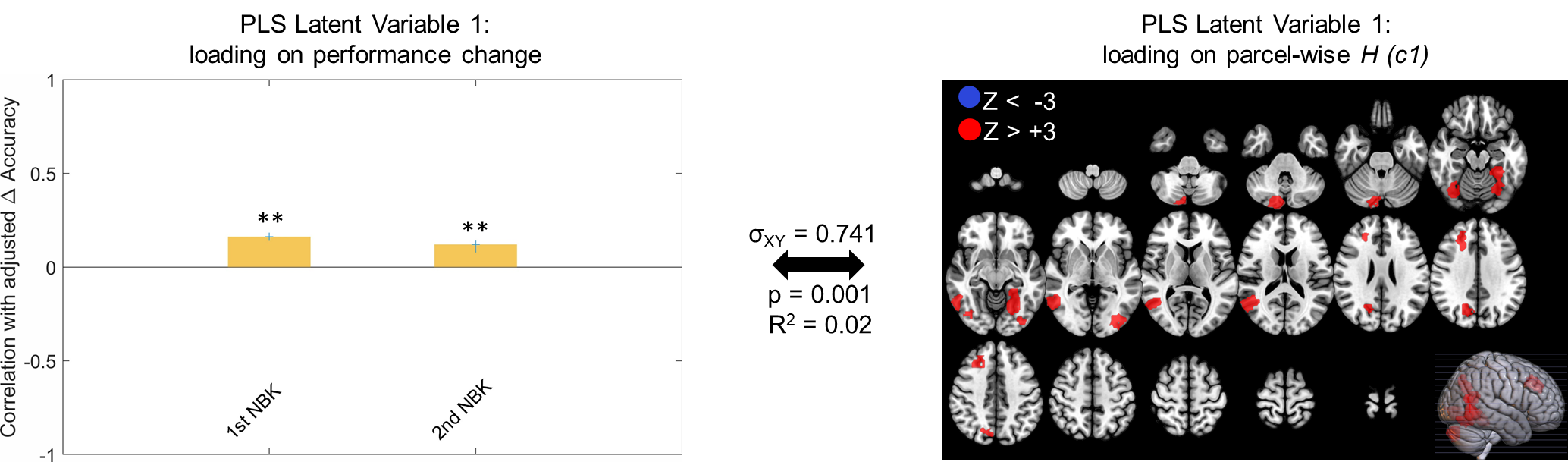


**Figure S11.** The primary latent variable from Behavioral PLS relating adj. ΔAccuracy in the NBK task to parcel-wise *H* estimated as *c*1 in WLMF analysis. Red parcels (total of 9) in the right panel show Bootstrap ratio Z_BR_ values above +3 and there are no blue parcels with Z_BR_ < −3, indicating exclusively positive direction for the H-to-adj. ΔAccuracy association. Cross-block covariance (σ_XY_) shows the proportion of covariance between the left and right panel explained by this LV, and the p value is calculated from a permutation test for the eigenvalue for this LV.

CAST task PLS results: Using the first cumulant (*c1*) of the WLMF to estimate the Hurst Exponent (*H*) instead of DFA yielded generally similar results in the PLS for the CAST study. Specifically, a pattern of higher *c1* across the brain was related to more improvement in the CAST task from run1 to run6, adjusted for performance in run1. On the behavioral side of the PLS’s LV1, the loading for run 3 became non-significant (see Figure S12; this run instead loaded on the second LV of the PLS, which was non-significant (p = .198) so is not included in the results). The spatial pattern in brain *H* (*c1)* from the primary latent variable was moderately correlated with the original *H* (DFA) analysis (r = .473, p<.001). The Z-thresholded maps were not overlapping, however. These are shown for brain *c1* latent variable in Figure S12, (5 parcels had Z>+3, 0 parcels had Z<-3; none of these 5 parcels were among the 4 parcels with Z>+3 in the DFA-based original n-back PLS result in Fig 5).


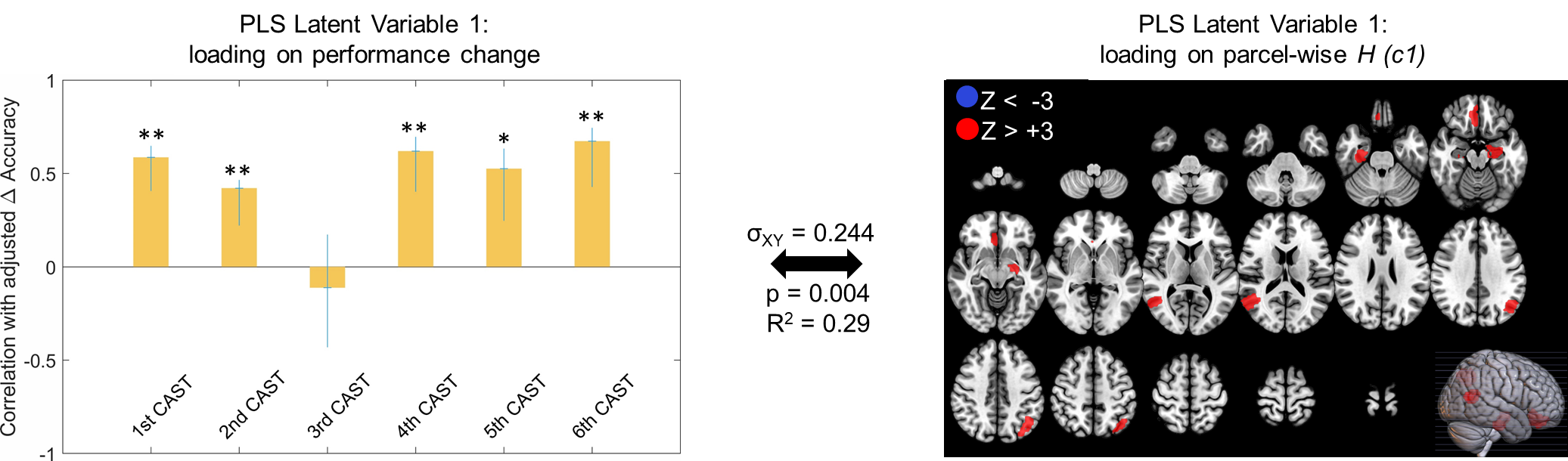


**Figure S12.** The primary latent variable from Behavioral PLS relating adj. ΔAccuracy in the CAST task to parcel-wise *H* estimated as *c*1 in WLMF analysis. All red parcels (total of 5) in the right panel show Bootstrap ratio Z_BR_ values above +3 and there are no blue parcels with Z_BR_ < −3, indicating exclusively positive direction for the H-to-adj. ΔAccuracy association. Cross-block covariance (σ_XY_) shows the proportion of covariance between the left and right panel explained by this LV, and the p value is calculated from a permutation test for the eigenvalue for this LV.

**Supplementary section 4.2. Higher order WLMF cumulants.**

We also quantified the second and third order cumulants (*c*2 and *c*3) from the WLMF analysis . Our analysis of the higher-order quadratic and cubic cumulants (i.e., *c*_2_ and *c*_3_) showed that these non-linear components had values close to zero for both the second order and 3^rd^ order cumulants across brain parcels in all runs of the three datasets (See Table S1), demonstrating a lack of non-linear scaling in these data.

|  | ***c*2: Mean (SD) [min, max])** | ***c*3: Mean (SD) [min, max])** |
| --- | --- | --- |
| **1^st^ DNB** | -.049 (.013) [-.308, .222] | -.003 (.005) [-.178, .133] |
| **Video** | -.041 (.011) [-.246, .178] | -.003 (.004) [-.156, .109] |
| **2^nd^ DNB** | -.050 (.012) [-.316, .242] | -.004 (.005) [-.191, .125] |
|  |  |  |
| **1^st^ NBK** | -.023 (.015) [-.223, .187] | -.005 (.005) [-.135, .105] |
| **2^nd^ NBK** | -.021 (.015) [-.222, .188] | -.006 (.005) [-.139, .107] |
|  |  |  |
| **1^st^ CAST** | -.064 (.020) [-.434, .403] | -.006 (.010) [-.321, .182] |
| **2^nd^ CAST** | -.067 (.020) [-.412, .368] | -.004 (.006) [-.297, .183] |
| **3^rd^ CAST** | -.068 (.021) [-.447, .419] | -.004 (.008) [-.314, .203] |
| **4^th^ CAST** | -.065 (.016) [-.414, .390] | -.004 (.007) [-.336, .176] |
| **5^th^ CAST** | -.071 (.026) [-.440, .417] | -.005 (.006) [-.337, .209] |
| **6^th^ CAST** | -.070 (.022) [-.456, .375] | -.006 (.014) [-.326, .206] |

**Table S1.** The second- and third-order cumulants from the WLMF fit to the fMRI data across the three datasets. SD is the standard deviation of whole-brain average cumulant value between participants. Min and max inside square brackets are minimum and maximum values observed across all brain parcels of all participants.

**Supplementary section 5. Non-adjusted change in performance.**

The adjustment of ΔAccuracy was motivated by three points. First, our hypothesis was about predicting who will improve their performance between participants who are initially performing at the same level (figure 2). To be close to this hypothetical scenario, albeit statistically, we adjusted the ΔAccuracy by regressing out the initial performance to make the measure linearly independent of the initial performance. The second reason can be argued based on the data and the regression to the mean component of Δperformance. Specifically, non-adjusted Δperformance is negatively correlated with baseline performance (DNB: r = -.280, p = .037; NBK: r = -.460, p <.001; CAST: r = -.716, p < .001) which is largely due to regression to the mean (i.e., starting lower allows for more room for increased performance). As such, without regressing out the baseline performance, the relationship between brain *H* and change in performance will capture an amalgam of variance due to regression to the mean and true Δperformance, while adjusted ΔAcc will capture portion of Δperformance that is independent of the regression to the mean. A third reason is based on the general neuroimaging discussion point related to the benefit of using brain data rather than purely behavioral measures. Adjusting for baseline performance means our *H* findings are explaining unique variance for practice effects independent of initial task performance. Therefore, if for example the goal is forecasting future task performance, a model will likely gain additional predictive power by adding the fMRI *H* as a predictive feature over and above previous task performance. Nevertheless, in this section we assessed the PLS regressions between parcel-wise *H* and ΔAccuracy without regressing out the baseline accuracy from ΔAccuracy. These results are shown in Figures S13-S15 for each dataset detailed below:

Dual n-back task: A pattern of higher *H* across the brain was related to greater improvement (ΔA’) in the DNB task from run1 to run2, not adjusted for performance in run1. The resulting brain LV was very highly correlated with the brain LV using adj. ΔA’ in the original analysis (r = .967, p <.001). The Z-thresholded brain parcels are shown in Figure S13, where 9 parcels had Z>+3. All 5 parcels with Z>+3 in the original results (Figure 3) were among the 9 parcels in the non-adjusted analysis.


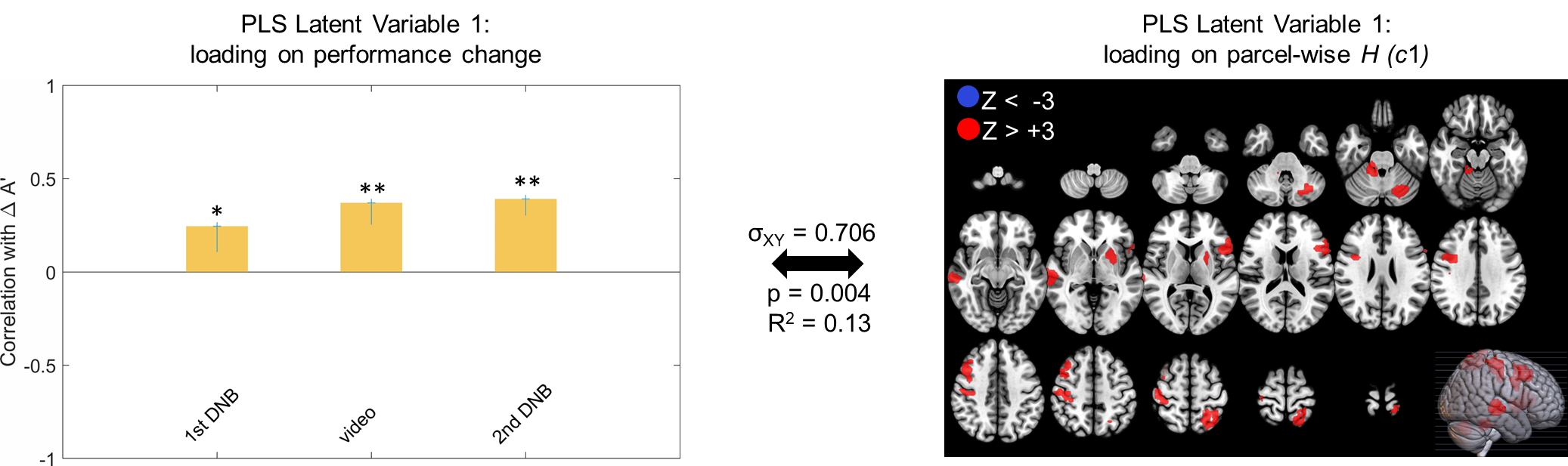


**Figure S13.** The primary latent variable from Behavioral PLS relating ΔA’ (non-adjusted) to parcel-wise *H* in the DNB experiment. All red parcels (total of 9) in the right panel show Bootstrap ratio Z_BR_ values above +3 and there are no blue parcels with Z_BR_ < −3, indicating exclusively positive direction for the H-to-ΔA’ association. Cross-block covariance (σ_XY_) shows the proportion of covariance between the left and right panel explained by this LV, and the p value is calculated from a permutation test for the eigenvalue for this LV.

N-back task: There were no significant latent variables in the PLS regression relating *H* across the brain to greater improvement in the NBK task from run1 to run2, not adjusted for performance in run1. Figure S14 shows the primary LV in this analysis which has p = .198 from the permutation test. This non-significant result, compared to Figure 4 results where adj. ΔAcc is used, could be due to mixing regression to the mean variance and true practice effects from the non-adjusted ΔAccuracy.


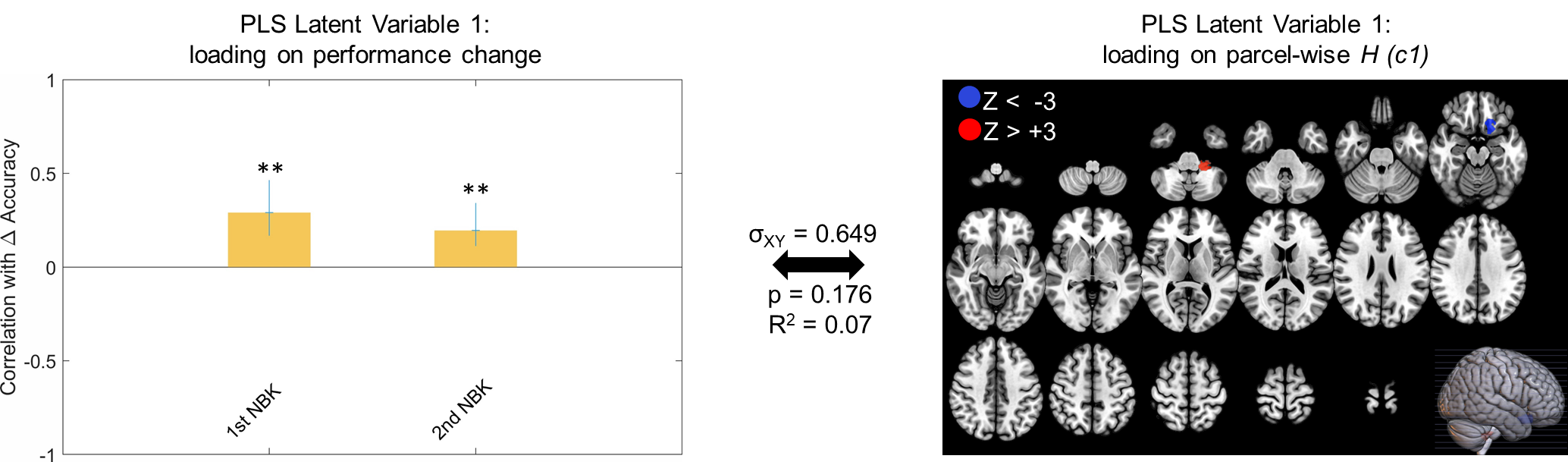


**Figure S14.** The primary latent variable from Behavioral PLS relating ΔAccuracy (non-adjusted) in the NBK task to parcel-wise *H*. Error bars in left panel show 95% confidence intervals as indicated by bootstrapping, which also yield the Z_BR_ values in the right panel (1 red, 1 blue). The p-value is calculated from a permutation test for the eigenvalue for this LV, and shows that this primary latent variable is not significantly different from the null distribution (p = .176, N.S.).

Choose-and-solve task: A pattern of higher *H* across the brain was related to greater improvement in the CAST task from run1 to run6, not adjusted for performance in run1. The resulting brain LV was highly correlated with the brain LV using adj. Δ Accuracy in the original analysis (r = .673, p <.001). The Z-thresholded brain parcels are shown in Figure S15, where 4 parcels had Z>+3. Despite the high correlation between the brain LVs in the continuous form, the Z>+3 parcels in this PLS did not overlap with the Z>+3 parcels from the original results (Figure 5).


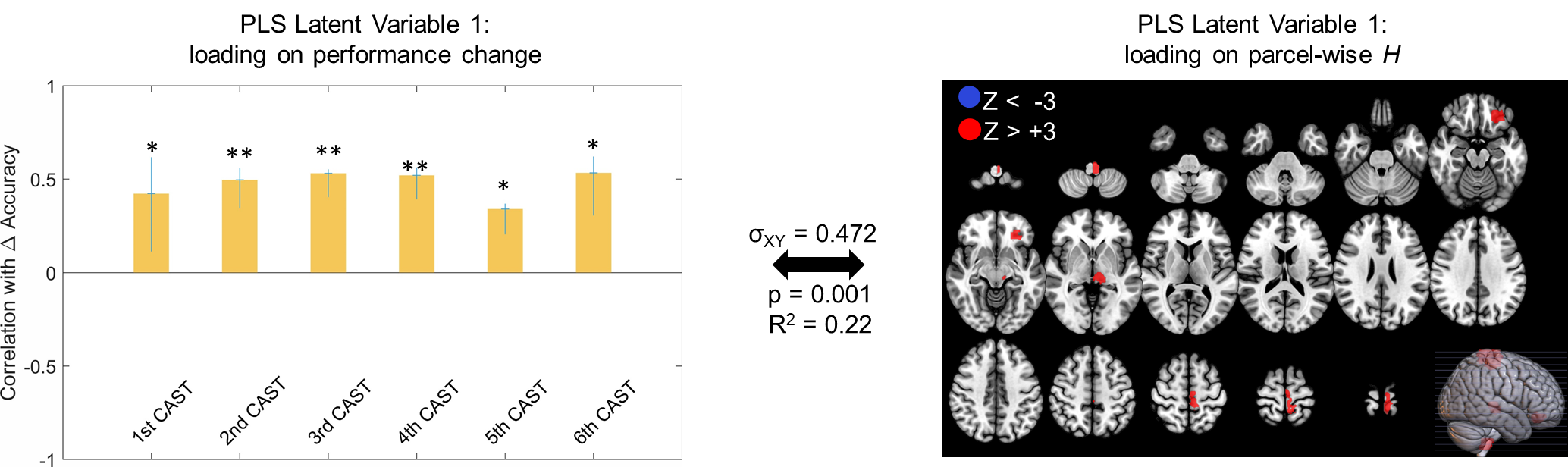


**Figure S15.** The primary latent variable from Behavioral PLS relating ΔAccuracy (non-adjusted) in the CAST task to parcel-wise *H*. All red parcels (total of 4) in the right panel show Bootstrap ratio Z_BR_ values above +3 and there are no blue parcels with Z_BR_ < −3, indicating exclusively positive direction for the H-to- Δ Accuracy association. Cross-block covariance (σ_XY_) shows the proportion of covariance between the left and right panel explained by this LV, and the p-value is calculated from a permutation test for the eigenvalue for this LV.
